# Ovipositional responses of tortricid moths to sugars, salts and neem oil

**DOI:** 10.1038/s41598-024-51972-1

**Published:** 2024-01-19

**Authors:** Carles Amat, Rajendra Prasad, César Gemeno

**Affiliations:** 1 University of Lleida-Agrotecnio-CERCA Center, Lleida, Spain; 2 ICAR-KVK Ramanagara, University of Agricultural Sciences, Bangalore, India

**Keywords:** Ecology, Physiology

## Abstract

Oviposition is essential in the life history of insects and is mainly mediated by chemical and tactile cues present on the plant surface. Oviposition deterrents or stimulants can modify insect oviposition and be employed in pest control. Relatively few gustatory oviposition stimuli have been described for tortricid moths. In this study the effect of NaCl, KCl, sucrose, fructose and neem oil on the number of eggs laid by *Cydia pomonella* (L.), *Grapholita molesta* (Busck) and *Lobesia botrana* (Dennis & Schifermüller) was tested in laboratory arenas containing filter papers loaded with 3 doses of a given stimulus and solvent control. In general, salts increased oviposition at the mid dose (10^2^ M) and sugars reduced it at the highest dose (10^3^ mM), but these effects depended on the species. Neem oil dramatically reduced the number of eggs laid as the dose increased, but the lowest neem oil dose (0.1% v/v) increased *L. botrana* oviposition relative to solvent control. Our study shows that ubiquitous plant chemicals modify tortricid moth oviposition under laboratory conditions, and that neem oil is a strong oviposition deterrent. The oviposition arena developed in this study is a convenient tool to test the effect of tastants on the oviposition behavior of tortricid moths.

## Introduction

Oviposition behavior is fundamental in the life history of oviparous animals. In phytophagous insects, female oviposition choices have a profound impact on larval fitness when they are restricted to the plant chosen by their mothers^1^. If the mother oviposits on a suboptimal host the larval may experience low fitness or die^2^. In addition, egg location determines susceptibility to natural enemies and environmental stressors such as insolation and desiccation, and the number of larvae per plant or plant part could affect larval fitness through competition, so females may also evaluate previous oviposition in the host in order to increase larval fitness^1^.

Female Lepidoptera (moths and butterflies) assess host suitability using different sensory modalities^1^. Olfaction and vision play a major role before landing, but once on the plant females have access to additional information, mainly non-volatile chemicals sensed through gustation (also referred to as taste or contact chemoreception), and physical parameters of the plants such as their shape and size^3–5^. Most of the plant chemo- and mechano-information is located on its cuticle^6^. In addition, internal chemical cues may become available to the insect where the cuticle breaches by action of its mouthparts, legs or ovipositor. Gustatory sensory neurons are typically housed inside microscopic cuticular projections (i.e., sensilla) which in the Lepidoptera, as in most insects, are located on the legs, cephalic appendages, the anterior sections of the digestive system and the ovipositor^7^. Tactile and gustatory information are often obtained simultaneously, either because mechano and gustatory sensilla co-occur in the same body location or because mechano and gustatory sensory neurons are housed in the same sensillum^8,9^. Just by walking on the plant females gather chemical information passively through their legs. In addition, female butterflies reportedly drum on the plant substrate with their forelegs to gather additional chemical information from the plant, and female moths and butterflies drag their ovipositor over the plant substrate, potentially to sample its chemical composition^3,10^.

Chemical oviposition stimulants and deterrents, both olfactory and gustatory, play different roles in pest management^11^. In push–pull strategies, deterrents can push the pest away from the crop while stimulants can be used to attract them to alternative sinks^12^. Oviposition marking pheromones, those that reduce larval competition, have been used successfully to reduce Tephritid fly populations^13^. Oviposition is also relevant in the control of insects of medical importance, such as mosquitoes^14^. Synthetic chemicals, such as modern insecticides, can also change oviposition behavior^15^. Oviposition cards to monitor eggs in species which eggs are hard to find could provide a very useful monitoring tool^16^.

Leafroller moths (Lepidoptera: Tortricidae) are economically important fruit pests^17^. Females lay eggs on the plant surface and the larvae feed on internal plant tissues (i.e., fruit, twigs, etc.). There are, however, relatively few oviposition stimuli described for tortricids. Foster and Howard^18^ found that organic-solvent extracts of host plants stimulated oviposition in *Epiphyas postvittan*a (Walker) (Lepidoptera: Tortricidae), and Grant et al.^19^ tested oviposition response of *Choristoneura fumiferana* (Clemens) (Lepidoptera: Tortricidae) to a series of aliphatic carboxylic acids. Rid et al.^20^ showed that oleanoic acid, the most abundant compound on the skin of grapes, stimulated oviposition of European grapevine moth (EGVM; *Lobesia botrana* Dennis & Schiffermüller). We have described the response of gustatory receptor neurons from antennae and labial palps of adult males and females of the Codling moth (CM; *Cydia pomonella* L.), the Oriental fruit moth (OFM; *Grapholita molesta* Busck) and the EGVM, the most economically important tortricid pests in apple, peach and grapevine worldwide, respectively^17,21^. The gustatory neurons responded to sugars (sucrose and fructose) and salts (KCl, NaCL)^22^.

In the present study we wanted to determine if the compounds that stimulated the gustatory neurons can also influence behavior. We chose oviposition instead of feeding behavior because there is no conclusive evidence that adults of these species, which have relatively short mouthparts, feed under natural conditions^22^. We also tested neem oil, an essential oil derived from the tissues of the neem tree, *Azadirachta indica* (A. Juss.), which is a strong insect repellent^23^. To perform multiple-choice oviposition tests under laboratory conditions a convenient oviposition arena was developed.

## Results

### Oviposition arenas

Two aspects that had to be considered in the design of the oviposition arenas were: (1) choosing a suitable oviposition substrate material, and (2) discouraging oviposition on the remaining areas of the arena. Several oviposition arena arrangements were tried before settling on a definitive one. Our first choice of oviposition substrate was waxed kitchen paper because it is used in our rearing facility. Stimulus was applied with an atomizer on the waxed paper, but upon drying the highest sugar concentrations droplets became solid and being hygroscopic their consistency varied from crystalline to sticky depending on ambient humidity. On the filter paper the stimulus distributed evenly and did not form droplets, making it a suitable oviposition substrate. In addition, our preliminary observations indicated that females laid as many eggs on untreated filter paper as they did on waxed paper, so filter paper was chosen as the oviposition test substrate for the final tests.

**Figure 1:**
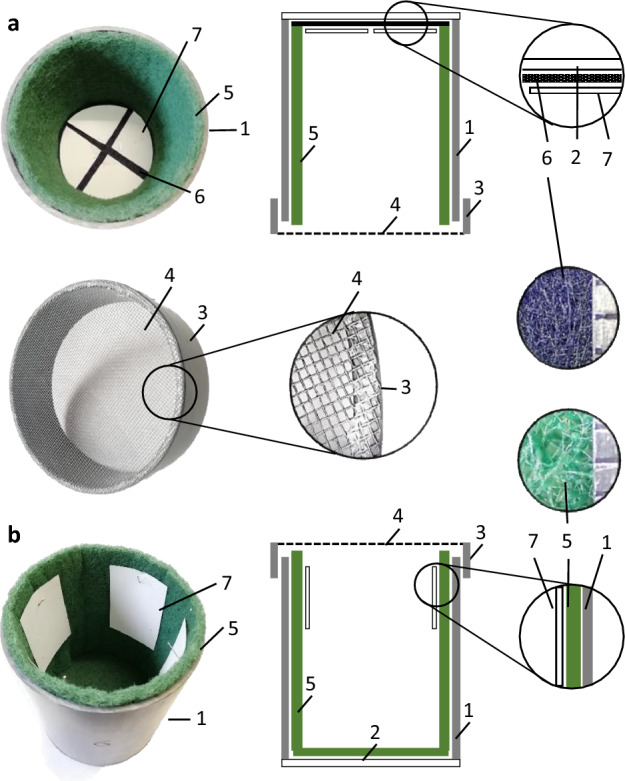
Oviposition arenas. PCV-pipe sections (1) were stopped with polymethyl methacrylate (2) and closed on the other side with a lid made of a short section of PVC (3) covered with nylon mosquito screen (4). The walls were lined with kitchen scourer (5) or black felt (6) to discourage oviposition (grey marks indicate 1 mm each). The four oviposition filter papers (7) were placed either a) on the ceiling in the EGVM (*L. botrana*) arenas, or b) on the walls in the OFM (*G. molesta*) and CM (*C. pomonella*) arenas, to maximize egg laying on the target substrate.

To concentrate oviposition on the test substrate the remaining of the arena must discourage females from egg laying, so in our first setup the oviposition cage walls were lined with black felt. EGVM and CM laid most of the eggs on the filter paper pieces attached to the felt, but OFM laid many eggs (11%, N = 11 arenas) on the felt, so it was replaced by kitchen scourer, which has a significantly more intricate surface (Fig. 1), and this reduced significantly the number of eggs laid by OFM outside the filter paper. Yet, with this setup, both OFM and EGVM laid a significant number of eggs on the lid (30% and 11%, N = 10 and 9 arenas, respectively), which at this initial stage was covered with polyester organza as in the rearing facility. The organza was replaced with nylon mosquito screen and now OFM and CM laid most of the eggs on the filter paper (97% and 99.9%, N = 10 and 8 arenas, respectively), but then EGVM laid too many eggs on the mosquito screen (18%, N = 10 arenas). Because we had observed that EGVM had a propensity to lay eggs on the ceiling of the cages, regardless of the material, we inverted the vertical position of the EGVM arenas and placed the filter paper on the ceiling while the lid was at the bottom, and with this last modification EGVM laid 88% of eggs on the target substrate (N = 4 arenas). As a final improvement, we lined the ceiling of the EGVM arenas with black felt to detect any eggs that were laid just outside the filter paper.

### Effect of tastants on oviposition

For each species and stimulus combination between 72 and 80 females were tested in groups of 4–5 in between 15 and 16 arena replications. CM laid between 11 and 70 eggs per female (36.82 ± 0.75, mean ± SEM), followed by OFM which laid between 12 and 70 eggs (34.05 ± 0.72, mean ± SEM) and EGVM which laid between 2 and 62 eggs (24.82 ± 0.75, mean ± SEM) (Supplementary Fig. S1).

The percentage of EGVM, OFM and CM females that had mated (according to spermatophore dissection) was 94, 90 and 96%, respectively (N = 50). Dead or morbid females at the end of the test occurred in 17 out of 229 arenas (Supplementary Table S1) with percentages of mortality per species (mean ± SEM) of 6.24 ± 2.74%, 0.53 ± 0.32% and 0.54 ± 0.33 for OFM, EGVM and CM, respectively. The highest morbidity/mortality occurred in the sucrose OFM arenas (15%). Mortality/morbidity did not appear to affect oviposition since the number of eggs deposited did not decrease as it increased (Supplementary Fig. S2).

The selected statistical model contained all main factors (stimulus, concentration and species) and 2nd- and 3rd-order interactions (Table 1a). The factor with the highest deviance (35% of total deviance) was the interaction between stimulus and concentration, followed by species (23%), and by the interaction between species and stimulus (15%) (Table 1b).

**Table 1:** Effect of stimulus, concentration and species on the number eggs laid by the females.

a							
Model type	AIC	Number of parameters	DF residual deviance	Residual deviance	LRT *p* value		
Null	6260	0	915	2872	–		
Main effects	5910	9	906	2504	< 0.001		
Main effects and 2nd-order interactions	5456	35	880	1998	< 0.001		
Main effects, 2nd- and 3rd-order interactions	5386	59	856	1880	< 0.001		

b							
Model term	DF	Deviance	DF residual deviance	Residual deviance	Pr (> Chisq)	% Dev.	Cum. Dev.
Null			915	2872			
Stimulus:Concentracion	12	344.55	880	1998	< 0.001	34.73	34.73
Species	2	225.53	913	2647	< 0.001	22.73	57.47
Species:Stimulus	8	146.67	898	2358	< 0.001	14.79	72.25
Species:Stimulus:Concentracion	24	118.11	856	1880	< 0.001	11.91	84.16
Concentracion	3	117.29	906	2504	< 0.001	11.82	95.98
Stimulus	4	24.94	909	2622	< 0.001	2.51	98.50
Species:Concentracion	6	14.91	892	2343	0.021	1.50	100.00

(a) Model comparison. The GLM model containing two- and three-order interactions of the three parameters was preferred according to Akaine information criterion (AIC) and likelihood ratio test (LRT). (b) Analysis of deviance of the selected model showing the contribution of each model parameter ordered by decreasing deviance. % Dev. = percentage of total deviance. Cum. Dev. = cumulative % Dev.

**Figure 2:**
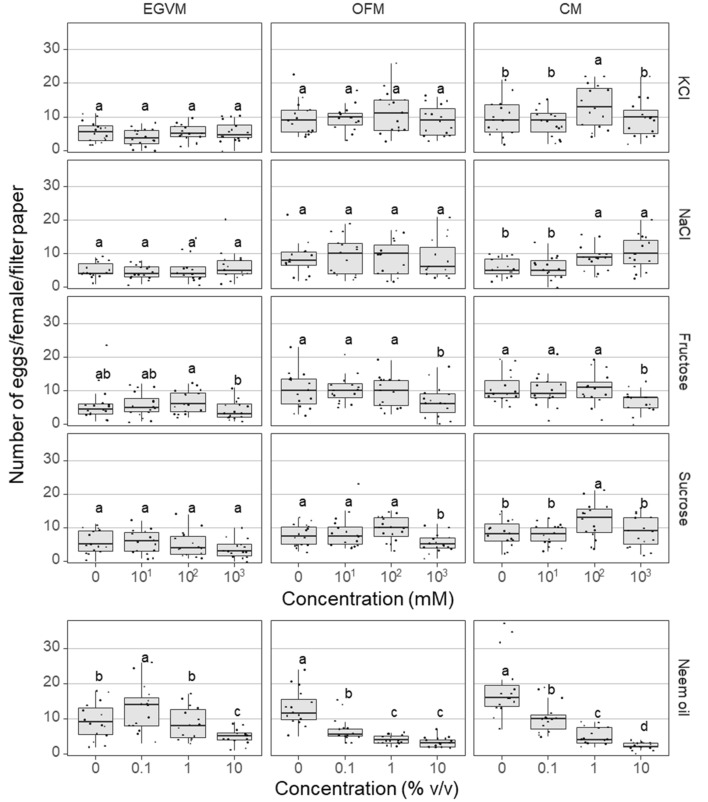
Effect of stimulus and concentration on the number of eggs laid by female EGVM (*L. botrana*), OFM (*G. molesta*) and CM (*C. pomonella*) on filter papers loaded with different concentrations of salts, sugars or neem oil. Within each plot, a different letter among treatments indicates significant differences (Tukey´s test, *P* < 0.05). Box plots show median (horizontal line), first and third quartile (box) and 1.5*inter-quartile range (vertical bars).

Pairwise comparisons of individual model parameters revealed that EGVM laid fewer total eggs than the other two species, that fructose and KCl received more eggs than the other three stimuli, and that the number of eggs decreased with increased concentration (Fig. 2, Supplementary Table S2). This general trend depended on the compound and species tested (Supplementary Table S3). Fewer eggs were laid on neem oil than on the other 4 stimuli in CM and OFM (except for NaCl in CM), while the opposite was true in the EGVM (more eggs in neem oil arenas than in the other treatment arenas in this species) (Fig. 2, Supplementary Table S3). Neem oil showed the strongest effect with concentration, whereas salts and sugars affected oviposition only at the highest dose (sugars inhibiting and NaCl stimulating it) (Fig. 2, Supplementary Table S3). Despite being the strongest inhibitor in all three species, the lowest neem oil dose stimulated EGVM oviposition above the control level (Fig. 2, Supplementary Table S4).

## Discussion

Convenient oviposition substrate for laboratory tests include plastic, glass, metal (e.g.,^24^), or paper (e.g.,^19^) because many moths tend to oviposit on smooth surfaces^1,16^. However, under our experimental conditions the non-porous substrate (waxed paper) was inconvenient due to the formation of hygroscopic droplets when using the highest sugar concentration. Because insects sense minute variations in surface texture^25,26^ that could affect moth oviposition, we opted for a porous substrate material (filter paper) that absorbed the stimulus and did not introduce physical artifacts. The number of eggs that females laid on the filter paper at the end of the study was similar to what has been reported in other studies^20,27,28^, supporting the suitability of filter paper as oviposition substrate for these species. We also found that kitchen scourer is more efficient than felt or similar materials to discourage oviposition (e.g.,^19,24,29^). The final oviposition cage configuration in our study was optimal in terms of number of eggs laid as well as in the handling aspects of insects and stimuli.

The observation that EGVM preferred to lay eggs on the underside was unexpected and required a specific cage setup for this species. Rid et al.^20^ found that EGVM laid 72% of the eggs on the ceiling of small (5-cm diameter) glass plates, but because in their study the ceiling was treated with stimuli it cannot be concluded that EGVM had an innate tendency to lay eggs on the ceiling in their setup. Markeiser et al.^16^ (Fig. 5 in^16^) made an interesting observation regarding the location and shape of the substrate on oviposition by EGVM and *Eupoecilia ambiguella* (Hübner) (another tortricid grape pest). Both species preferred to lay eggs on a convex-shaped (over a concave-shaped) surface if it was placed on the ceiling of the arena, while it was avoided when on the floor, except when it was shaded, in which case it was preferred by *E. ambiguella*. Female insects utilize the three-dimensional (i.e., spatial) characteristics of the substrate to make oviposition choices. Foster et al.^30^ showed that *E. postvittana* females prefer to lay eggs on leaves that have a simulated midrib vein and that they lay more eggs on dummy plants that have a denser leaf array. Stem diameter and distance between leaves and stem affect oviposition choices in the noctuid *Busseola fusca* (Fuller) (Lepidoptera: Noctuidae)^31^. *Plodia interpunctella* (Hübner) (Lepidoptera: Pyralidae) prefers to oviposit on spherical glass beads over flat surfaces or glass beds of other shapes, and there is an optimal oviposition bead size for this species^32^. In choice tests *Drosophila suzukii* (Matsumura) (Diptera: Drosophilidae) shows a preference for the smallest oviposition substrate diameter, and this ability is reduced after years of laboratory rearing using flat oviposition surfaces^26^. The moths *Yponomeuta cagnagellus* (Hübner) (Lepidoptera: Yponomeutidae), *C. fumiferana*, and EGVM prefer to lay eggs on artificial substrates, either cylindrical or spherical, that most resemble their oviposition hosts^24,33,34^. Our test species are adapted to lay eggs on plant parts that change with the season. EGVM lays eggs on the bracts of flowering grape bunches in the first generation, but on following generations it lays on the fruit^21^. OFM lays eggs mainly on shoots or leaves^35^ and CM oviposits mainly on leaves and less on branches or fruits^29,36^. Future tests should explore the effect of shape, size and texture on the oviposition of these tortricid moths.

Oviposition arenas provide an oversimplified environment where many of these natural cues are either absent or greatly modified. Yet, they offer a convenient tool to screen many gustatory compounds under controlled conditions, and provide information useful in further tests under more natural conditions, in a similar way as the wind tunnel and olfactometer serve to screen volatile compounds. In addition, our simplified oviposition arenas allow for dose–response curves, which are a lot more informative than single-dose tests, as shown by the increased oviposition of EGVM to the lowest neem oil concentration.

Salts are a limiting resource to most organisms, including phytophagous insects. Adults of some lepidopteran species actively seek salts on different sources, including minerals, animal faeces, urine or tears^37^. Ingesting salts may help achieve nutritional needs^38^, but whether female moths use salts to select larval hosts is practically unexplored. Maher et al.^39^ showed that KCl deterred oviposition in EGVM, while we found the opposite effect. Laboratory and field tests with Na-enriched host plants showed no oviposition effects in two butterfly species, even when the concentration was potentially toxic to the larvae^37^. Salt detection is critical in the feeding biology of haematophagous insects, and the concentration-dependent effect observed is non-linear^40^. Certainly, when salt levels are toxic females may choose not to oviposit, and this may be the reason why salts deter mosquito oviposition^41^.

Fruits are rich in sugars and this may serve to signal larval host quality to prospective ovipositing females, yet females do not always have fruits available for oviposition and it is not clear how important are sugars to female moths which larvae feed on green plant tissues. Whereas CM larvae feed only on the fruit of their host plants^42^, OFM larvae feed on new shoots on the first generation (when fruits are still absent), and on fruits on subsequent generations^43^. EGVM larvae feed on flowers, green berries or ripe berries of grapevines as they become available on the first, second and third moth generations, respectively^21^. Thus, sugars alone may not be essential oviposition cues for these species, at least not in every generation. Many moth species use nectar as an adult food source, but there is no inconclusive evidence in our test species: EGVM visits the flowers of tansy, *Tanacetum vulgare* L. but whether it consumes its nectar needs to be confirmed^44^. Host-plant sugars alter the oviposition behavior of CM and could play a role in its control^45,46^. Tarsal contact-chemoreceptor sensilla of EGVM are sensitive to fructose and sucrose, and females lay more eggs on substrates which have been treated with fructose and glucose, so perhaps these sugars may be of special importance when ovipositing on fruits^39^. We have found that males and females of the three test species detect sugars and salts with the gustatory sensilla located on their labial palp and antenna, so these compounds are probably relevant to some extent^22^. The effect of sugars on the oviposition of other moth species is scarce. Derridj et al.^47^ showed that *Ostrinia nubilalis* (Hübner) (Lepidoptera: Crambidae) females prefer to lay eggs on *Zea mays* (L.) with a higher content in free sugars (glucose, sucrose, fructose) induced by maleic hydrazide. Since this increase happened on the internal plant tissues it is not clear how the females had access to this information.

Neem oil contains at least 100 biologically active compounds and affects diverse aspects of insect behavior and physiology, including oviposition^23,48^. Seljåsen and Meadow^49^ showed a 50% reduction in the number of eggs laid by *Mamestra brassicae* (L.) (Lepidoptera: Noctuidae) on cabbage leaves treated with 0.5% neem oil. Bruce et al.^50^ report dramatic reductions in the number of eggs laid by noctuid and pyralid moths on the plant, already from the lowest neem oil dose tested. These tests, as ours and several others, involve contact between females and neem oil. There is physiological evidence that neem oil is sensed by gustatory sensilla since it, or its related terpenoids, appear to inhibit the electrophysiological response of gustatory sensilla in the labial palps of termites^51^, and the tarsal sensilla of *Plutella xyllostella* (L.) (Lepidoptera: Plutellidae)^52^. However, neem oil contains volatile compounds, so it is possible that its effect on oviposition is mediated by olfaction instead of, or in addition to, taste. Indeed, insects from diverse clades, including leafhoppers^53^, mosquitoes^54^ and lepidoptera^55^, respond to neem oil volatiles. 

It is worth noticing the increased oviposition of EGVM at the lowest neem oil dose (0.1%). Grant et al.^19^ report a very interesting observation, somewhat related to ours, where nonanoic acid (a 9-carbon carboxylic acid) loaded on filter paper in a petri dish strongly stimulated oviposition by *C. fumiferana* at the lowest of two doses (78 nmol/cm^2^), whereas it strongly inhibited it at the 10-times higher dose (780 nmol/cm^2^), and the opposite effect occurred with decanoic acid. Additionally, it is possible that EGVM females could not detect the repellent ingredients at the lowest concentration, but they were still able to detect oviposition stimulants present in the neem oil. This finding highlights the importance of testing more than one dose of test stimuli in chemostimulation tests.

Besides neem oil, other oils affecting moth oviposition include the egg extracts of EGVM and related species, which contain fatty acids that deter female oviposition and thus may reduce intra- and inter-specific competition^56^. Methanol extracts of *Nicotiana tabacum* (L.), which likely contain non-polar compounds, deter OFM oviposition when applied to peach twigs^57^. Oils from grape skin, specifically oleanolic and uralic acids, are shown to stimulate EGVM oviposition^20^. Oils may stimulate oviposition on moth species that feed on oil-rich sources, as Nansen and Phillips^58^ have shown for *P. interpunctella* using 17 types of oil-treated whole-wheat kernels.

Since non-polar compounds appear to be so relevant in insect oviposition, it would be valuable to explore the electrophysiological response of gustatory sensilla to non-polar stimuli to determine which body appendage bears the sensory neurons, as we have shown with polar stimui (salts and sugars) in the antennnae and palpi in the three test species^22^. However, unlike olfaction, recording taste electrophysiological responses to non-polar compounds requires the use of non-polar solvents or adding surfactants to the saline solution which may alter the normal neuron responses, so progress in this area is slow^8^. Detailed behavioral observations show that antennal and foreleg tapping and ovipositor dragging are involved in the context of moth oviposition^3,10,31,59,60^. In some Lepidoptera species the female has more taste sensilla on the prothorathic legs possibly allowing them to analyze the surface of the leaves on which they will lay eggs^8^.

Our study increases the list of oviposition-modifying chemical compounds described for tortricidae and shows that neem oil is a potent oviposition inhibitor. Making a suitable oviposition arena is essential to test different compounds simultaneously, and testing several doses was fundamental to reveal subtle differences, such as the increase in oviposition with the lowest neem oil dose in EGVM. Further research is needed to identify a larger number of compounds with potential gustatory effects, and to determine the mechanism of sensory detection, specifically for non-polar compounds. Performing tests under more natural conditions would be required to demonstrate the potential of these tastants in pest control, as has been shown with sugars in the CM^17^.

## Material and methods

### Insects

Larvae were reared on a semi-artificial diet^22^ at 25 °C under a 16:8 light:dark photoperiod. OFM and EGVM pupae were sexed and kept in 1-L containers provided with 10% sucrose in water in separate male and female environmental chambers and the adults were collected every 1–3 days. CM adults emerged in the larval cages and were collected daily. 1- to-3-day-old males and females were mixed in a ratio of 1.5:1, respectively, starting at least 5 h before the onset of the scotophase. On the following day, the now 2 to-4-day-old females were placed in the oviposition arenas. No food or drink (i.e., sugar water solution) was provided in the oviposition arenas. Mating status was checked after the test.

### Chemical stimuli

Water soluble stimuli (salts and sugars) were diluted in deionized water. Three concentrations of each stimulus (10, 100 and 1000 mM) were prepared in 5-ml aliquots. A sample of the same water stock was also kept as a control. Neem oil from cold pressed seeds (Batch no 9344732, Gonaturals, Vivere Gmbh, Hamburg, Germany) was diluted in absolute ethanol to obtain 0.1, 1 and 10% v/v dilutions. A sample of the same ethanol stock was also kept as a control. Dilution and solvent vials were kept at − 20 °C.

### Oviposition arenas

The oviposition arenas (Fig. 1) were adapted from the mating boxes used for insect rearing. A 138-mm-high × 117-mm-internal diameter (i.e., 1.48 L) section of solid grey PVC pipe was stopped in one end with a clear polymethyl methacrylate (PMMC) plate glued into it. The other end of the cylinder was covered with a lid made with a 40-mm section of a 125-mm inner diameter solid gray PVC pipe with grey nylon mosquito screen (mesh size = 1 mm) glued on it.

The walls were lined with 5-mm thick green kitchen scourer, protruding 5-mm outside the cylinder to ensure a good sealing with the mosquito screen lid. The blind side of arena was lined with either green kitchen scourer (CM and OFM) or 2-mm-thick black felt (EGVM). The oviposition substrate consisted of filter paper (Whatman No.1, Global Life Science Solutions Operations UK Ltd, Buckinghamshire, UK). In the CM and OFM arenas four 50-mm × 75-mm (37.5cm^2^ each) filter-paper pieces were pinned onto the scourer lining the walls, with the long-side vertical and spaced 30 mm from each other, 10-mm below the mosquito screen lid and 53-mm from the floor of the cage. In the EGVM arenas four triangular filter paper pieces were cut from a 110-mm radius disc (19.63 cm^2^ each) and were stapled to the black felt lining the end of the arena, leaving 10-mm between filter papers. CM and OFM arenas stood upside up, with the mosquito screen lid on top, while EGVM arenas stood upside down, with the nylon screen lid on the floor, and the filter papers on the ceiling. This difference in arena orientation maximized the number of eggs laid on the filter papers relative to the other arena surfaces (i.e., kitchen scourer, nylon screen or felt).

### Experimental procedure

The 5-ml aliquots were defrosted and refrozen one or more times to load samples throughout the experiment. Filter paper pieces with the treatment name lightly written with pencil were laid on a plastic screen to facilitate evaporation. Test solutions were applied using micropipetes with disposable plastic tips, wetting the paper completely and as evenly as possible. The CM and OFM filter papers received 0.5 ml test solution, and the LOB papers 0.26 ml, resulting in similar stimulus quantity per unit area in both filter paper formats (Supplementary Table S5). A different pipette tip was used for each compound, the control treatment was loaded first and then the test stimuli, from the lowest to the highest concentration using the same pipette tip for a given compound. Latex gloves were exchanged when handling filter papers of different stimuli. Filter papers were left at room temperature until they dried out, and then were kept inside plastic bags at 4 °C until used, but for no longer than 15 d.

Females were introduced in the test arenas at least 5 h before the onset of the scotophase. The arenas were placed in the same environmental chamber (Supplementary Fig. S3) where the colony was kept, at 24.5 ± 1 °C. A 18-W, 17-cm-diameter household ceiling LED fixture (Brass & Fittings S.L, Zaragoza, Spain) was placed 27-cm above the area were the arenas where. A fluorescent light bulb attached to the door on the other side of the environmental chamber and 80-cm away from the experimental arenas provided additional chamber illumination (Philips, Master TL-D 36W/840 REFLEX Eco, Koninklijke Philips N.V., Amsterdam, Netherlands). White filter paper placed on a shelf 20 cm underneath the arenas reflected light to the arenas from below. The amount of light entering the arenas varied according to their position on the shelf. CM and OFM arenas received between 110 and 1390 lx and LOB arenas received between 80 and 103 lx, inside and near the mosquito-screen lid respectively. Arena positions were randomized between days to account for positional effects.

The number of eggs laid on the filter papers was recorded 24 h after females were released in the oviposition arenas. Females were placed in ethanol for later determination of mating status by means of spermatophore dissection (N = 50 females/species). For cleaning, scourer and felt were submerged in 60 °C tap water for 20 min and then were rubbed thoroughly and let dry before reuse. The mosquito screen was cleaned with a paper towel soaked in 75% ethanol, and the cages were rinsed in water.

### Statistics

Statistical analyses and plots were created with R software v4.2.2.^61^. The number of eggs per female was estimated by dividing the number of eggs in a filter paper by the number or females placed in the arena (4–5). Generalized lineal models (GLM) with Poisson error distribution were used to analyze the effect of species, stimulus and concentration. Model selection started from the simplest model containing no main effects, then main factors and interactions were added sequentially. The models were compared with each other with the likelihood ratio test (LRT) and the Akaike information criterion (AIC), preferring the model with the lower AIC of pairs that were significantly different by LRT. The model with the best fit was used to conduct pairwise comparison between relevant groups of significant factors. Pairwise comparisons used the Tukeys´s test in the package “emmeans”.

### Guideline statement

All procedures of the experiments, including the collection and use of organisms, complied with relevant institutional, national, and international guidelines and legislation.

### Supplementary Information

Supplementary Information.

## Data Availability

Raw data and R-script for statistical analysis are available online at 10.34810/data588.
