# Supplementary material for: Ovipositional responses of tortricid moths to sugars, salts and neem oil
Source: Sci Rep. 2024 Jan 19;14:1677. doi: 10.1038/s41598-024-51972-1 (PMC10799066; doi:10.1038/s41598-024-51972-1)
Supplement: Supplementary file 1 — Supplementary Information. [file 41598_2024_51972_MOESM1_ESM.pdf]

Ovipositional responses of tortricid moths to sugars, salts and neem oil

Carles Amat, Rajendra Prasad, César Gemeno

Supplementary material

Table S1 Mortality and morbidity of *L. botrana* (EGVM), *G. molesta* (OFM) and *C. pomonella* (CM) in the oviposition test.

Species	Stimulus	Number of females	Number morbid/dead	% morbid/dead	Number of arenas with morbid/dead
EGVM	KCl	80	0	0.00	0
	NaCl	75	1	1.33	1
	Fructose	80	0	0.00	0
	Sucrose	75	1	1.33	1
	Neem oil	75	0	0.00	0
OFM	KCl	75	1	1.33	1
	NaCl	74	7	9.46	5
	Fructose	74	4	5.41	2
	Sucrose	80	12	15.00	5
	Neem oil	80	0	0.00	0
CM	KCl	75	1	1.33	1
	NaCl	72	0	0.00	0
	Fructose	73	0	0.00	0
	Sucrose	72	1	1.39	1
	Neem oil	75	0	0.00	0

Table S2 Number of eggs laid by *L. botrana* (EGVM), *G. molesta* (OFM) and *C. pomonella* (CM) in the oviposition test. Observed data and model estimates. Different letters indicate significant differences among levels for each parameter (species, stimulus and concentration, Tukey´s test, P<0.05)

	Observed	Estimated	
Species	Mean ± SEM	Mean ± SEM	
EGVM	6.00 ± 0.24	5.74 ± 0.14	b
OFM	8.51 ± 0.28	8.15 ± 0.17	a
CM	9.17 ± 0.32	8.51 ± 0.18	a
Stimulus			
KCl	8.34 ± 0.38	7.94 ± 0.21	a
NaCl	7.28 ± 0.34	7.04 ± 0.20	b
Fructose	8.13 ± 0.35	7.84 ± 0.21	a
Sucrose	7.40 ± 0.32	7.04 ± 0.20	b
Neem oil	8.25 ± 0.47	6.99 ± 0.21	b
Concentration			
Control	8.99 ± 0.38	8.45 ± 0.20	a
Low	7.99 ± 0.31	7.62 ± 0.19	b
Medium	8.26 ± 0.33	7.80 ± 0.19	ab
High	6.29 ± 0.29	5.83 ± 0.17	c

Table S3 Number of eggs laid by *L. botrana* (EGVM), *G. molesta* (OFM) and *C. pomonella* (CM) in the oviposition test for different treatment combinations (observed data and model estimates). (A) species within stimulus, (B) stimulus within species, and (C) concentration within stimulus. Different letters for each parameter in the first column indicate significant differences among the levels of the parameter in the second column for each parameter of the first column (Tukey´s test P<0.05).

A

Species	Stimulus	Observed Mean ± SEM	Estimated Mean ± SEM	
EGVM	KCl	5.05 ± 0.34	4.99 ± 0.28	b
	NaCl	5.38 ± 0.43	5.35 ± 0.30	b
	Fructose	5.67 ± 0.50	5.58 ± 0.30	b
	Sucrose	5.03 ± 0.44	4.96 ± 0.29	b
	Neem oil	8.97 ± 0.73	8.46 ± 0.39	a
OFM	KCl	9.97 ± 0.65	9.92 ± 0.41	a
	NaCl	8.75 ± 0.68	8.74 ± 0.38	ab
	Fructose	9.35 ± 0.63	9.22 ± 0.40	a
	Sucrose	7.84 ± 0.49	7.67 ± 0.35	b
	Neem oil	6.78 ± 0.62	5.87 ± 0.32	c
CM	KCl	10.23 ± 0.72	10.08 ± 0.41	a
	NaCl	7.70 ± 0.53	7.48 ± 0.36	b
	Fructose	9.53 ± 0.59	9.37 ± 0.40	a
	Sucrose	9.30 ± 0.58	9.19 ± 0.39	a
	Neem oil	9.10 ± 1.04	6.88 ± 0.39	b

B

Stimulus	Species	Observed Mean ± SEM	Estimated Mean ± SEM	
KCl	EGVM	5.05 ± 0.34	4.99 ± 0.28	b
	OFM	9.97 ± 0.65	9.92 ± 0.41	a
	CM	10.23 ± 0.72	10.08 ± 0.41	a
NaCl	EGVM	5.38 ± 0.43	5.35 ± 0.30	c
	OFM	8.75 ± 0.68	8.74 ± 0.38	a
	CM	7.70 ± 0.53	7.48 ± 0.36	b
Fructose	EGVM	5.67 ± 0.50	5.58 ± 0.30	b
	OFM	9.35 ± 0.63	9.22 ± 0.40	a
	CM	9.53 ± 0.59	9.37 ± 0.40	a
Sucrose	EGVM	5.03 ± 0.44	4.96 ± 0.29	c
	OFM	7.84 ± 0.49	7.67 ± 0.35	b
	CM	9.30 ± 0.58	9.19 ± 0.39	a
Neem oil	EGVM	8.97 ± 0.73	8.46 ± 0.39	a
	OFM	6.78 ± 0.62	5.87 ± 0.32	b
	CM	9.10 ± 1.04	6.88 ± 0.39	b

C

Stimulus	Concentration	Observed Mean ± SEM	Estimated Mean ± SEM	
KCl	0	8.35 ± 0.73	8.14 ± 0.43	ab
	10	7.20 ± 0.62	6.77 ± 0.40	b
	100	9.87 ± 0.93	9.24 ± 0.47	a
	1000	7.96 ± 0.70	7.78 ± 0.42	ab
NaCl	0	6.51 ± 0.56	6.34 ± 0.38	b
	10	6.67 ± 0.66	6.38 ± 0.38	b
	100	7.64 ± 0.67	7.47 ± 0.41	ab
	1000	8.29 ± 0.78	8.14 ± 0.43	a
Fructose	0	9.00 ± 0.81	8.79 ± 0.44	a
	10	8.65 ± 0.68	8.43 ± 0.43	a
	100	9.00 ± 0.67	8.88 ± 0.44	a
	1000	5.87 ± 0.57	5.76 ± 0.36	b
Sucrose	0	7.33 ± 0.53	7.21 ± 0.40	a
	10	7.50 ± 0.60	7.35 ± 0.40	a
	100	8.89 ± 0.74	8.37 ± 0.44	a
	1000	5.89 ± 0.59	5.55 ± 0.36	b
Neem oil	0	13.70 ± 1.12	13.18 ± 0.55	a
	0.1	9.91 ± 0.81	9.64 ± 0.46	b
	1	5.89 ± 0.54	5.62 ± 0.36	c
	10	3.50 ± 0.28	3.34 ± 0.28	d

Table S4 Number of eggs laid by *L. botrana* (EGVM)*, G. molesta* (OFM) and *C. pomonella* (CM) in the oviposition test (observed data and model estimates). Effect of concentration within species and species within stimulus. Different letters in each species by stimulus concentration indicate significant differences among concentrations (Tukey´s test P<0.05).

Stimulus	Species	Concentracion	Observed Mean ± SEM	Estimated Mean ± SEM	
KCl	EGVM	0	0.29 ± 0.02	0.29 ± 0.02	a
		10	0.19 ± 0.02	0.19 ± 0.02	b
		100	0.26 ± 0.02	0.26 ± 0.02	ab
		1000	0.27 ± 0.02	0.27 ± 0.02	a
	OFM	0	0.24 ± 0.02	0.24 ± 0.02	a
		10	0.25 ± 0.02	0.25 ± 0.02	a
		100	0.28 ± 0.03	0.28 ± 0.02	a
		1000	0.22 ± 0.02	0.22 ± 0.02	a
	CM	0	0.24 ± 0.02	0.24 ± 0.02	b
		10	0.20 ± 0.02	0.20 ± 0.02	b
		100	0.33 ± 0.02	0.33 ± 0.02	a
		1000	0.23 ± 0.02	0.23 ± 0.02	b
NaCl	EGVM	0	0.25 ± 0.02	0.25 ± 0.02	a
		10	0.23 ± 0.02	0.23 ± 0.02	a
		100	0.25 ± 0.02	0.25 ± 0.02	a
		1000	0.28 ± 0.03	0.28 ± 0.02	a
	OFM	0	0.25 ± 0.02	0.25 ± 0.02	a
		10	0.27 ± 0.03	0.27 ± 0.02	a
		100	0.24 ± 0.03	0.24 ± 0.02	a
		1000	0.24 ± 0.02	0.24 ± 0.02	a
	CM	0	0.20 ± 0.02	0.20 ± 0.02	b
		10	0.19 ± 0.02	0.19 ± 0.02	b
		100	0.28 ± 0.02	0.28 ± 0.02	a
		1000	0.33 ± 0.03	0.33 ± 0.02	a
Fructose	EGVM	0	0.25 ± 0.02	0.25 ± 0.02	ab
		10	0.25 ± 0.02	0.25 ± 0.02	ab
		100	0.31 ± 0.02	0.31 ± 0.02	a
		1000	0.19 ± 0.03	0.19 ± 0.02	b
	OFM	0	0.27 ± 0.03	0.27 ± 0.02	a
		10	0.28 ± 0.03	0.28 ± 0.02	a
		100	0.26 ± 0.03	0.26 ± 0.02	ab
		1000	0.19 ± 0.03	0.19 ± 0.02	b
	CM	0	0.28 ± 0.02	0.28 ± 0.02	a
		10	0.28 ± 0.03	0.28 ± 0.02	a
		100	0.27 ± 0.03	0.27 ± 0.02	a
		1000	0.17 ± 0.02	0.17 ± 0.02	b
Sucrose	EGVM	0	0.28 ± 0.02	0.28 ± 0.02	a
		10	0.28 ± 0.02	0.28 ± 0.02	a
		100	0.27 ± 0.03	0.27 ± 0.02	a
		1000	0.17 ± 0.02	0.17 ± 0.02	b
	OFM	0	0.25 ± 0.03	0.25 ± 0.02	ab
		10	0.27 ± 0.02	0.27 ± 0.02	a
		100	0.30 ± 0.02	0.30 ± 0.02	a
		1000	0.18 ± 0.02	0.18 ± 0.02	b
	CM	0	0.23 ± 0.02	0.23 ± 0.02	b
		10	0.22 ± 0.01	0.22 ± 0.02	b
		100	0.32 ± 0.02	0.32 ± 0.02	a
		1000	0.23 ± 0.03	0.23 ± 0.02	b
Neem oil	EGVM	0	0.26 ± 0.03	0.26 ± 0.02	b
		0.1	0.35 ± 0.02	0.35 ± 0.02	a
		1	0.24 ± 0.02	0.24 ± 0.02	b
		10	0.15 ± 0.02	0.15 ± 0.02	c
	OFM	0	0.47 ± 0.02	0.47 ± 0.02	a
		0.1	0.24 ± 0.01	0.24 ± 0.02	b
		1	0.16 ± 0.01	0.16 ± 0.02	c
		10	0.13 ± 0.01	0.13 ± 0.02	c
	CM	0	0.50 ± 0.02	0.50 ± 0.02	a
		0.1	0.28 ± 0.02	0.28 ± 0.02	b
		1	0.16 ± 0.02	0.16 ± 0.02	c
		10	0.07 ± 0.01	0.07 ± 0.02	d

Table S5 Stimulus source and concentration per filter paper

Stimulus	CAS	Sigma	Ref.	Concentration mM (sugars and salts), v/v (neem oil)	Amount in filter paper μg/cm^2
KCl	7447-40-7	P3911-500G-M	P3911	10	9.94
				100	99.38
				1000	993.75
NaCl	7647-14-5	S7653-1KG	S7653	10	7.79
				100	77.90
				1000	779.01
Fructose	50-48-7	F0127-1KG	F0127	10	24.02
				100	240.15
				1000	2401.53
Sucrose	57-50-1	S9378-1KG	S9378	10	45.63
				100	456.29
				1000	4562.86
Neem oil	-	-	9344732	0.1	1.20
				1	12.00
				10	120.00

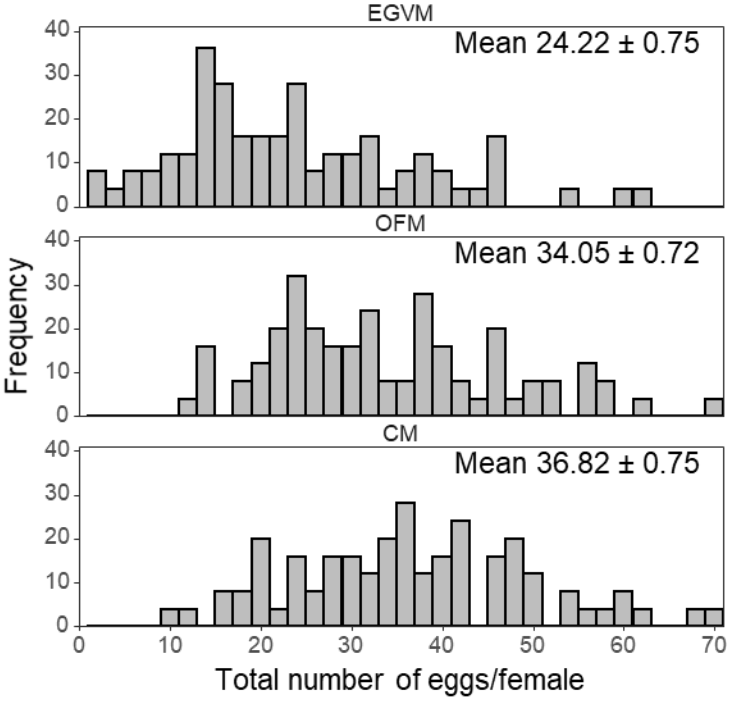

Fig. S1 Distribution of the number of eggs laid by individual *L. botrana* (EGVM), *G. molesta* (OFM) and *C. pomonella* (CM) females in the oviposition test.

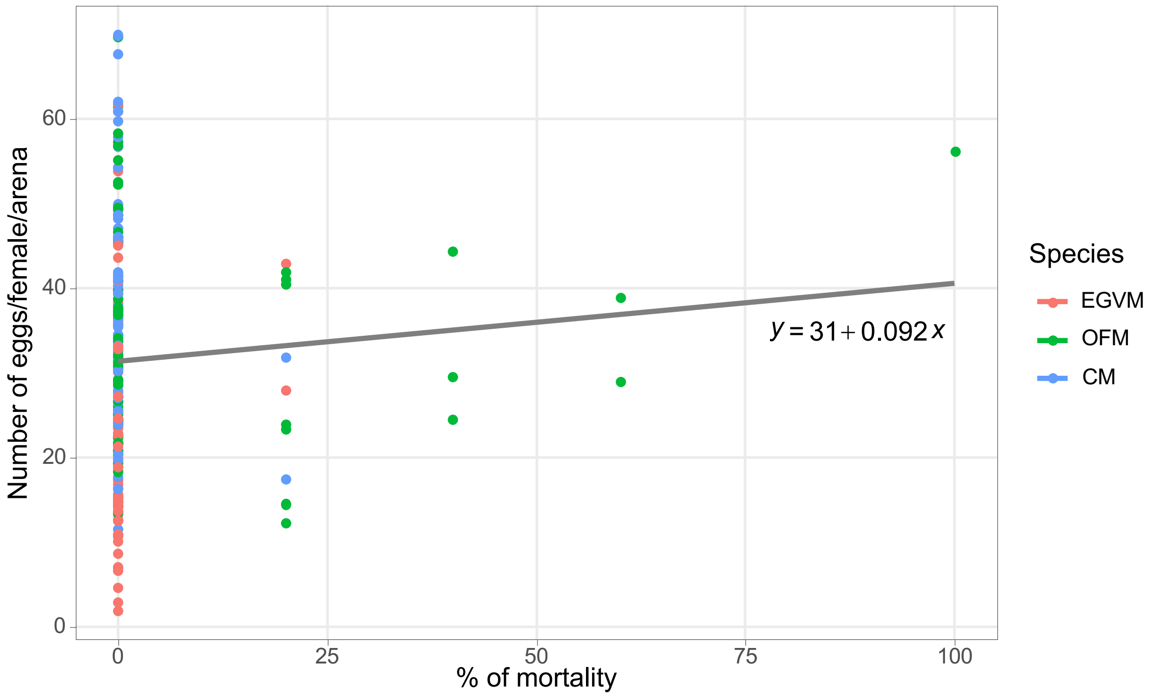

Fig. S2 Correlation between the number of eggs laid by *L. botrana* (EGVM), *G. molesta* (OFM) and *C. pomonella* (CM) and the number of females dead in the oviposition tests.

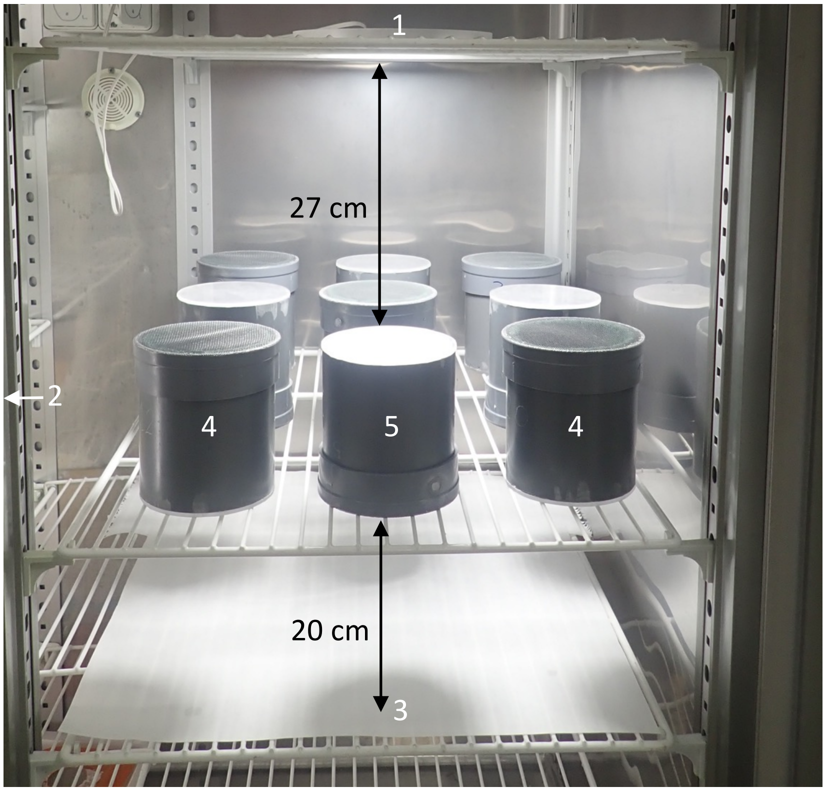

Fig. S3 Element distribution in the environmental chambers during oviposition experiments. (1) household ceiling LED fixture, (2) fluorescent light bulb (out of sight, 80cm away from the oviposition arenas attached to the door), (3) white filter paper reflecting light from 1, (4) OFM (*G. molesta*) and CM (*C. pomonella*) arenas and (5) EGVM (*L. botrana*) arenas.
